# Genome-wide screening of sex-biased genetic variants potentially associated with COVID-19 hospitalization

**DOI:** 10.3389/fgene.2022.1014191

**Published:** 2022-10-24

**Authors:** Yu-Si Luo, Wei Li, Yi Cai, Jingxuan Zhang, Hongsheng Gui, Ke Zhang, Zhong-Shan Cheng

**Affiliations:** ^1^ Department of Emergency, The Affiliated Hospital of Guizhou Medical University, Guiyang, China; ^2^ The Key and Characteristic Laboratory of Modern Pathogenicity Biology, School of Basic Medical Sciences, Guizhou Medical University, Guiyang, China; ^3^ Department of Cardiovascular, The Affiliated Hospital of Guizhou Medical University, Guiyang, China; ^4^ Guangdong Key Laboratory of Regional Immunity and Diseases, Department of Pathogen Biology, Shenzhen University School of Medicine, Shenzhen, China; ^5^ Hubei Key Laboratory of Embryonic Stem Cell Research, School of Basic Medical Sciences, Hubei University of Medicine, Shiyan, China; ^6^ Center for Health Policy and Health Services Research, Behavioral Health Services and Department of Psychiatry, Henry Ford Health System, Detroit, MI, United States; ^7^ Center for Applied Bioinformatics, St. Jude Children’s Research Hospital, 262 Danny Thomas Hospital, Memphis, TN, United States

**Keywords:** GWAS, single nucleotide polymorphisms, COVID-19 hospitalization, European population, sex-biased

## Abstract

Sex-biased difference in coronavirus disease 2019 (COVID-19) hospitalization has been observed as that male patients tend to be more likely to be hospitalized than female patients. However, due to the insufficient sample size and existed studies that more prioritized to sex-stratified COVID-19 genome-wide association study (GWAS), the searching for sex-biased genetic variants showing differential association signals between sexes with COVID-19 hospitalization was severely hindered. We hypothesized genetic variants would show potentially sex-biased genetic effects on COVID-19 hospitalization if they display significant differential association effect sizes between male and female COVID-19 patients. By integrating two COVID-19 GWASs, including hospitalized COVID-19 patients vs. general population separated into males (case = 1,917 and control = 221,174) and females (case = 1,343 and control = 262,886), we differentiated the association effect sizes of each common single nucleotide polymorphism (SNP) within the two GWASs. Twelve SNPs were suggested to show differential COVID-19 associations between sexes. Further investigation of genes (n = 58) close to these 12 SNPs resulted in the identification of 34 genes demonstrating sex-biased differential expression in at least one GTEx tissue. Finally, 5 SNPs are mapped to 8 genes, including rs1134004 (*GADD45G*), rs140657166 (*TRIM29* and *PVRL1*), rs148143613 (*KNDC1* and *STK32C*), rs2443615 (*PGAP2* and *TRIM21*), and rs2924725 (*CSMD1*). The 8 genes display significantly differential gene expression in blood samples derived from COVID-19 patients compared to healthy controls. These genes are potential genetic factors contributing to sex differences in COVID-19 hospitalization and warranted for further functional studies.

## Introduction

The severe acute respiratory syndrome coronavirus 2 (SARS-CoV-2) was first emerged in Wuhan, China, in October 2019, and then it was spread to almost all countries in the world. Until now, SARS-CoV-2 induced coronavirus disease 2019 (COVID-19) has led to ∼6.32 million deaths worldwide according to COVID-19 Dashboard from Johns Hopkins University (Dong et al., 2020). Genetic factors have been suggested to contribute to different variation of COVID-19 severity, and two large-scale genome-wide association studies (GWASs) related to the severe COVID-19 phenotype (The Severe Covid-19 GWAS Group, 2020; Pairo-Castineira et al., 2021) have uncovered several loci in human genome predisposing to COVID-19 severity. Significant single nucleotide polymorphisms (SNPs) have been reported to associate with the risk of severe COVID-19. These SNPs are rs11385942 located in the 3p21.31 region harboring the genes *SLC6A20*, *LZTFL1*, *CCR9*, *FYCO1*, *CXCR6*, and *XCR1*, rs10735079 mapped to a gene cluster comprising *OAS1, OAS2, *and *OAS3*, and three other SNPs, including rs74956615, rs2109069, and rs2236757, mapped to *TYK2*, *DPP9*, and *IFNAR2*, respectively (Pairo-Castineira et al., 2021). In addition, increasing studies have been reported that COVID-19 leads to more severe symptoms and higher mortality in men than in women (Alwani et al., 2021). A large-scale cohort comprising 17 million European adults discovered that male sex was more predisposing to COVID-19 induced death (hazard ratio 1.59) (Williamson et al., 2020). A recent study claimed that male tends to have weaker immune responses, particularly T cell response against SARS-CoV-2 infection compared to female. However, the study was based on a dataset with relatively small sample size (n < 100) and did not adjust for confounding factors in the analyses, the conclusion of which was criticized by researchers (Takahashi et al., 2020; Shattuck-Heidorn et al., 2021; Takahashi et al., 2021; Danielsen et al., 2022). Therefore, whether or how sex differences contribute to COVID-19 severity/hospitalization is still not clear. Furthermore, previously published COVID-19 GWASs mainly focused on both sexes altogether or considered males and females separately (Tu et al., 2020; Viveiros et al., 2021). Recently, several COVID-19 risk SNPs have been reported by sex-stratified GWASs, such as rs17763742, rs4443214, and rs35477280, which are three risk SNPs located in the famous 3p21.31 region and are predominantly associated with COVID-19 in males (Cruz et al., 2022). However, due to the requirement of large sample sizes for both sexes, the determination of the interaction between SNPs and sexes with COVID-19 severity/hospitalization was hindered. As few genome-wide significant sex-biased COVID-19 SNPs have been reported, there is an urgent need to search for sex-biased genetic factors associated with COVID-19, given that substantial sex differences in inflammation, immunity, and aberrant renin-angiotensin system (RAS) activity have been observed in the pathogenesis of COVID-19. Many immune and inflammation association genes are X-linked, including but not limited to *TLR7*, *TLR8*, and *IRAK1* (Viveiros et al., 2021). Females have an increased IFN-α secretion, early virus sensing, and prompt antiviral response upon *TLR7* stimulation in dendritic cells. In addition, the *ACE2* gene resides in the Xp22.2 region of the X chromosome and is recognized as an escape gene. Females theoretically have a double dose of *ACE2*, which may compensate for SARS-CoV-2 mediated loss of membrane ACE2 and alleviate aberrant RAS activity and related cardiovascular diseases (Tu et al., 2020; Viveiros et al., 2021). To search for novel sex-biased loci, i.e., loci displaying significantly differential associations with COVID-19 hospitalization between sexes, we performed an integrative study of sex-biased genetic factors on COVID-19 by comparing male- and female-stratified COVID-19 hospitalization GWAS summary statistics from UK Biobank. Here the sex-biased variants represent variants showing differential association signals based on the Z-test between the effect sizes of each SNP from sex-stratified COVID-19 hospitalization GWASs. Genes close to these SNPs were evaluated for its potential sex-biased differential expression as well as its involvements in COVID-19.

## Materials and methods

Summary statistics of two COVID-19 hospitalization GWASs by males and females were obtained from GRASP (Thibord et al., 2022) (https://grasp.nhlbi.nih.gov/Covid19GWASResults.aspx) that were generated based on UK Biobank data. The two COVID-19 hospitalization GWASs were conducted on samples with European descent and designed by comparing hospitalized COVID-19 cases with not hospitalized COVID-19 patients for males and females individually (1,917 hospitalized COVID-19 males vs. 221,174 not hospitalized COVID-19 males; 1,343 hospitalized COVID-19 females vs. 262,886 not hospitalized COVID-19 females). We used the statistical method introduced by Thibord et al. (Thibord et al., 2022) to compare the difference of effect size beta for each common SNP (MAF >0.01, imputation score >0.6) between the two GWASs by calculating its delta Z-score (ΔZ-score) and its corresponding *p* value, the formulas of which are as follows:

$$\Delta Z-score=\frac{female.gwas.\beta -male.gwas.\beta }{\sqrt{\left({female.gwas.se}^{2}+male.gwas.{se}^{2}\right)}}$$

p=pnorm(−|ΔZ−score|)∗2

Common SNPs were kept with MAF >0.01 and imputation score >0.6 in both male- and female-stratified COVID-19 GWASs. The genome-wide significance threshold of ΔZ-score *p* values was set as *p* < 5.0 × 10^−8^; a relaxed *p*-value threshold for ΔZ-score *p* values, *p* < 5 × 10^−6^, was applied to select SNPs with suggestive evidence of sex-biased, differential COVID-19 associations. Only top SNPs passing the relaxed ΔZ-score *p*-value threshold and representing independent, differential COVID-19 association signals were selected for further evaluation.

Genes adjacent to the selected top SNPs were subject to sex-biased differential expression analysis in GTEx tissues (The GTEx Consortium, 2020) and differential gene expression (DGE) analysis between COVID-19 patients and healthy controls. We located genes within the 1-Mbp genomic window with one of the top SNP centered. To gain biological insights about these top SNPs and genes close to them, we determined whether these top SNPs are also cis-expression quantitative trait loci (cis-eQTL) in GTEx database (Oliva et al., 2020) and eQTLGen database (Võsa et al., 2018). We further evaluated whether these SNPs adjacent genes showing DGE between the sexes across GTEx tissues (Oliva et al., 2020). In detail, we downloaded summary statistics of DGE between sexes from GTEx portal (https://storage.googleapis.com/gtex_analysis_v8/sex_biased_genes_and_sbeqtl_data/GTEx_Analysis_v8_sbgenes.tar.gz) and checked the sex-biased DGE *p* values and its corresponding effect sizes for these genes located within a 1-Mbp window of the top SNPs showing sex-biased COVID-19 associations. To determine the fold change of median gene expression between sexes for these genes in GTEx tissue, we downloaded GTEx gene expression Transcripts Per Million (TPM) matrix and sex information for all samples from these links (TPM matrix: https://storage.googleapis.com/gtex_analysis_v8/rna_seq_data/GTEx_Analysis_2017-06-05_v8_RNASeQCv1.1.9_gene_tpm.gct.gz; tissue and sample information: https://storage.googleapis.com/gtex_analysis_v8/annotations/GTEx_Analysis_v8_Annotations_SubjectPhenotypesDS.txt; sample sex, age, and other clinical information: https://storage.googleapis.com/gtex_analysis_v8/annotations/GTEx_Analysis_v8_Annotations_SampleAttributesDS.txt). After extraction of TPMs for these SNP adjacent genes, we determined the fold change of median gene expression using the formula log2 (female/male). In addition, we queried these genes in COVID-19 expression database COVID19db, a gene expression database related to SARS-CoV-2 infection (Zhang et al., 2022), and determined whether these genes were differentially expressed in blood samples of COVID-19 patients compared to healthy controls (Thair et al., 2021) with one-way ANOVA.

## Results

**FIGURE 1:**
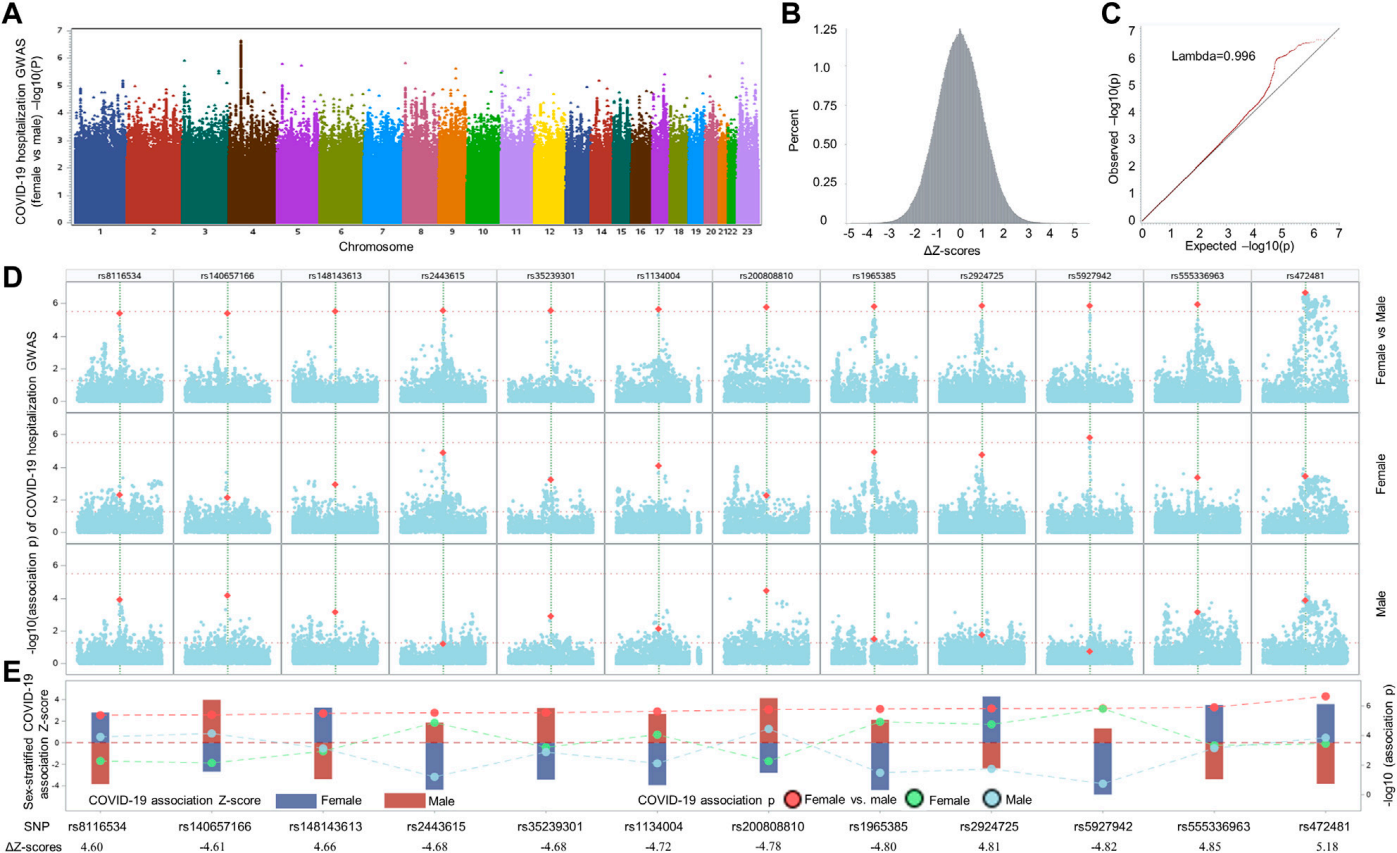
Comparative analysis of sex-stratified genome-wide association studies of hospitalized COVID-19 cases vs. general population. **(A)** Manhattan plot illustrating the comparison of effect size beta of each single nucleotide polymorphism (SNP) between the two COVID-19 hospitalization GWASs with European ancestry from UK Biobank. **(B)** Delta Z-score (ΔZ-score) histogram (see method section for the calculation of ΔZ-score). **(C)** QQ plot of ΔZ-score *p* values. **(D)** Local Manhattan plot covering a 1-Mbp region harboring each of 12 top SNPs (highlighted in dark yellow color) that were passed the nominal ΔZ-score *p* value threshold of *p* < 5 × 10^−6^ (upper panel), followed by another two local Manhattan plots demonstrating sex-stratified COVID-19 association signals. **(E)** Needle plot at the bottom illustrating the sex-stratified and sex-biased COVID-19 association signals. The sex-stratified SNP Z-scores are displayed according to the left *y*-axis for both sexes (female: dark blue bar; male: dark red bar). The SNP *p* values of sex-stratified association signals for female, represented by light green dots, and male, indicated by light blue dots, as well as the corresponding *p* values of ΔZ-scores, highlighted by red dots, are displayed in accordance to the right *y*-axis. To separate these *p* values and visualize the differences of Z-scores and association *p* values in both sexes, dashed lines are utilized to link and separate these dots representing association *p* values derived from sex-stratified analysis and ΔZ-score *p* values generated by differential Z-test between sexes. The ΔZ-score for each SNP is listed at bottom of panel E.

To search for SNPs displaying sex-biased association signals in hospitalized COVID-19 patients, we compared the association effect size beta of each SNP between two COVID-19 hospitalization GWASs stratified by sexes among samples with European ancestry, which were publicly available from GRASP COVID-19 database (Thibord et al., 2022). The two GWASs were conducted by comparing hospitalized COVID-19 with general population for males and females individually, adjusted by age and 10 principal components, with all samples from European ancestry. When considering the two GWASs individually, only one independent genome-wide significant SNP, rs13071258, mapped to the gene cluster of *SLC6A20*, *LZTFL1*, *CCR9*, *FYCO1*, *CXCR6*, and *XCR1*, with *p* = 9.97 × 10^−10^ and beta = 0.43, emerged from the male GWAS, while in the corresponding female GWAS, the SNP displayed moderate association signal with COVID-19 hospitalization (*p* = 4.12 × 10^−5^; beta = 0.34). Because the SNP demonstrated similar effect sizes, there was no significant difference in terms of ΔZ-score (value = −0.84; ΔZ-score *p* = 0.40). We further compared SNP association effect size beta of the two GWASs and determined the ΔZ-scores genome-wide, resulting in 12 independent, differential association signals (all ΔZ-score *p* values <5 × 10^−6^) between the female and male COVID-19 GWASs, including rs8116534, rs140657166, rs148143613, rs2443615, rs35239301, rs1134004, rs200808810, rs1965385, rs2924725, rs5927942, rs555336963, and rs472481 (Figure 1 and Table 1). The estimated skewness for the ΔZ-scores genome-wide is 0.016, suggesting the close normal distribution of ΔZ-scores. This is in line with the GWAS inflation lambda, which is 0.996, indicating weak inflation of the sex-biased GWAS based on ΔZ-score *p* values. Taken together, only one independent SNP emerged as genome-wide significant when considering the two sex-stratified GWASs individually, with the SNP does not show significant differential effect size between sexes; further genome-wide screening of sex-biased COVID-19 risk SNPs harvested 12 candidate SNPs.

**TABLE 1:** Top variants displaying sex-biased associations with COVID-19 hospitalization in UK Biobank.

Chr*	Position*	A1/A2^∧^		rsid	ΔZ-score *p* value	ΔZ-score	GWAS *p*		Z-score		Beta		Adjacent sex-biased DGE^#^
							Female	Male	Female	Male	Female	Male	
4	43,498,824	C	T	rs472481	2.2 × 10^−7^	5.18	3.6 × 10^−4^	1.4 × 10^−4^	3.57	−3.81	0.27	−0.24	
3	6,540,177	T	TT	rs555336963	1.2 × 10^−6^	4.85	4.7 × 10^−4^	7.2 × 10^−4^	3.50	−3.38	0.21	−0.17	*GRM7*
23	32,200,753	T	C	rs5927942	1.4 × 10^−6^	−4.82	1.5 × 10^−6^	0.18	−4.80	1.33	−0.21	0.03	
8	4,841,239	C	T	rs2924725	1.5 × 10^−6^	4.81	1.8 × 10^−5^	1.8 × 10^−2^	4.29	−2.36	0.20	−0.09	*CSMD1*
5	17,633,867	T	C	rs1965385	1.6 × 10^−6^	−4.80	1.2 × 10^−5^	3.3 × 10^−2^	−4.38	2.13	−1.49	0.52	*BASP1*
5	105,404,915	G	A	rs200808810	1.7 × 10^−6^	−4.78	5.4 × 10^−3^	3.6 × 10^−5^	−2.78	4.13	−0.15	0.18	
9	91,981,877	C	T	rs1134004	2.3 × 10^−6^	−4.72	8.8 × 10^−5^	7.6 × 10^−3^	−3.92	2.67	−0.19	0.11	*S1PR3*, *GADD45G*, *SHC3*, *SECISBP2*, *SEMA4D*, *C9orf47*
3	159,373,186	G	T	rs35239301	2.8 × 10^−6^	−4.68	6.2 × 10^−4^	1.3 × 10^−3^	−3.42	3.21	−0.25	0.19	*SCHIP1*
11	4,249,031	C	T	rs2443615	2.8 × 10^−6^	−4.68	1.4 × 10^−5^	6.3 × 10^−2^	−4.35	1.86	−3.64	0.96	*RRM1*, *STIM1*, *PGAP2*, *TRIM21*
10	134,578,626	T	C	rs148143613	3.2 × 10^−6^	4.66	1.2 × 10^−3^	7.4 × 10^−4^	3.25	−3.37	0.50	−0.44	*C10orf91*, *PWWP2B*, *KNDC1*, *LRRC27*, *STK32C*, *INPP5A*
11	119,728,423	A	C	rs140657166	4.0 × 10^−6^	−4.61	7.2 × 10^−3^	7.3 × 10^−5^	−2.69	3.97	−0.31	0.38	*TRIM29*, *OAF*, *THY1*, *PVRL1*, *AP000679.2*
20	18,068,955	C	T	rs8116534	4.2 × 10^−6^	4.60	5.3 × 10^−3^	1.2 × 10^−4^	2.79	−3.84	0.16	−0.19	*RBBP9*, *DZANK1*, *ZNF133*, *CSRP2BP*, *RRBP1*, *MGME1*, *OVOL2*, *POLR3F*, *SEC23B*

Notes: *: chromosomal position based on the human reference hg19 build.^#^: genes located within the 1-Mbp window where the SNP at the center were determined for sex-biased differential gene expression analysis by Olivia et al. (Oliva et al., 2020).

^ indicates the risk allele.

We further evaluated previously published COVID-19 risk SNPs showing genome-wide significant association signals in either females/males or both sexes published by Cruz et al. (Cruz et al, 2022) in the currently used UK Biobank COVID-19 hospitalization GWASs. We obtained summary statistics of nine genome-wide significant SNPs emerged in sex-unstratified or sex-stratified COVID-19 GWASs from Cruz and colleagues (Cruz et al., 2022). After performing Z-test to determine differential association signals between females and males for these SNPs, we only found three SNPs passed the ΔZ-score *p* value threshold of *p* < 0.05 (Supplementary Figure S1) in the data set from Cruz et al. (Cruz et al., 2022). In the currently used UK Biobank COVID-19 GWASs of females and males, none of these nine SNPs demonstrated differential association between sexes with COVID-19 (all ΔZ-score *p* > 0.1; Supplementary Figure S1). However, six out of these nine SNPs were nominally significant in the current UK Biobank sex-stratified COVID-19 GWASs, including these three SNPs (rs17763742, rs115679256, and rs35477280) located in the 3p21.31 region. We further conducted (LD) analysis between these three SNPs and the genome-wide significant SNP rs13071258 from the male only COVID-19 hospitalization GWAS from UK Biobank. We revealed that rs17763742 and rs13071258 are in complete LD (*R* ^2^ = 1; D’ = 1) in European population; the other two SNPs are in weak LD to rs13071258 in European population, with *R* ^2^ = 0.08 for rs13071258 vs. rs115679256 and *R* ^2^ = 0.43 for rs13071258 vs. rs17763742. We confirmed that most of these published COVID-19 SNPs by Cruz et al. (Cruz et al., 2022) are replicable in the current sex-stratified COVID-19 GWASs in either males or females. The lack of replication of sex-biased differential associations for these SNPs in current study may attribute to the relatively small sample sizes of current UK Biobank GWASs. In conclusion, in the current sex-biased COVID-19 GWAS with relatively small sample sizes, previously published genome-wide significant SNPs do not show significant difference of effect size between sexes, which is in line with the requirement of GWAS with large sample size to determine sex-biased COVID-19 risk SNPs.

To investigate the potential regulatory roles played by these top 12 SNPs emerged from sex-biased COVID-19 GWAS, we queried them in GTEx Portal (The GTEx Consortium, 2020) and eQTLGen (Võsa et al., 2018). We identified 4 cis-eQTLs; among them, rs140657166 on chromosome 6, is a cis-eQTL of *THY1* in cells-cultured fibroblasts, which is also close to *PVRL1* (alias *NECTIN1*) and *TRIM29*. In addition, another variant rs1134004, on chromosome 9, is strongly associated with *SEMA4D* expression among multiple GTEx tissues, including cells-cultured fibroblasts, artery-tibial, testis, breast-mammary tissue, and other tissues. eQTLGen also reported the SNP as eQTL for *SEMA4D* in blood samples. Additionally, rs8116534 is an eQTL of the non-coding gene *LINC00652* on chromosome 20 in muscle skeletal and ovary, and three brain tissues, including amygdala, hypothalamus, and cerebellum. One indel, rs555336963, which is a strong cis-eQTL of the RNA gene *AC069277* on chromosome 3 in multiple tissues (thyroid, spleen, pituitary, small intestine-terminal ileum, cells-EBV-transformed lymphocytes, colon-transverse, and esophagus-mucosa). Furthermore, rs5927942 is the only SNP located on chromosome X, but it is not an eQTL for any adjacent genes according to GTEx database and eQTLGen database. For other top SNPs, including rs472481, rs2924725, rs2443615, and rs1965385, they are not cis-eQTLs in both GTEx database and eQTLGen database; rs148143613, rs35239301, and rs200808810 are not included in GTEx database with no eQTL records for them in eQTLGen database.

**FIGURE 2:**
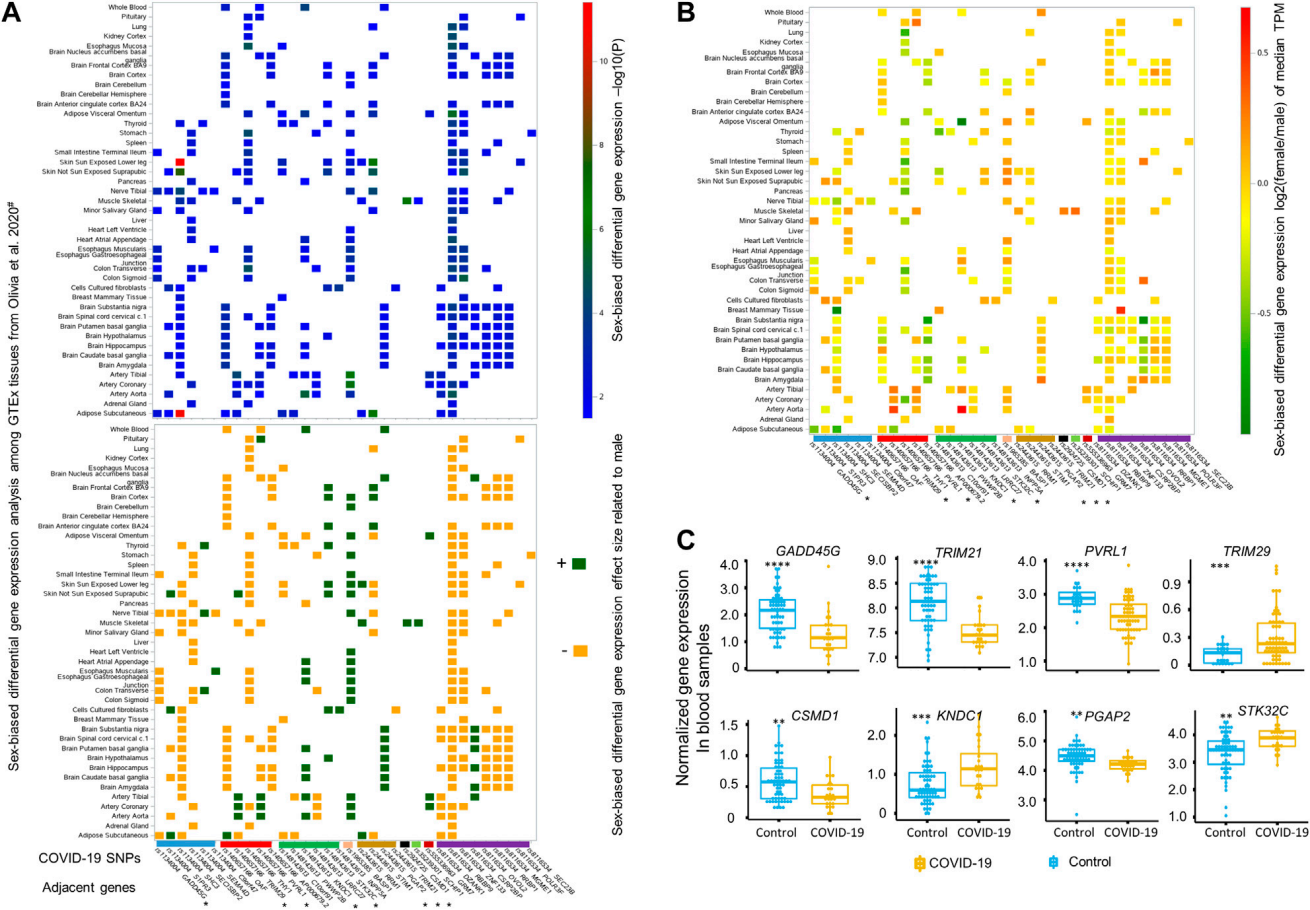
Differential gene expression analyses by sex or upon SARS-CoV-2 infection for genes close to these 12 top sex-biased COVID-19 risk SNPs. **(A)** Summary statistics of sex-biased differential gene expression analysis, including -log10(P) (top panel) and its corresponding direction of effect size (lower panel), for 34 sex-biased genes are illustrated based on the data generated by Olivia et al., across 43 different GTEx tissues (Oliva et al., 2020). The directions of effect size are represented by dark green (‘+’, higher expression in female) and dark yellow (‘−’, lower expression in female). The median gene expression fold change derived by comparing females to males for these 34 sex-biased genes is depicted in **(B)**, with a color bar on the right side indicating the magnitude of log2 (fold change) of median gene expression of Transcripts Per Million (TPM) between females and males. Among these 34 sex-biased genes, eight genes (marked with * in panel A and B) adjacent to five top sex-biased COVID-19 risk SNPs displaying up- or down-regulated expression **(C)** and also passed the multiple adjusted significance threshold of *p* < 0.015 in blood samples of COVID-19 patients (n = 62) compared to uninfected healthy controls (n = 23) (Thair et al., 2021), with statistical significance determined by one-way ANOVA test and denoted by **: *p* ≤ 0.01; ***: *p* ≤ 0.001; ****: *p* ≤ 0.0001.

We further examined protein codes genes located within a 1-Mbp genomic region with each of these top 12 COVID-19 SNPs located at the center. Based on the DGE analysis performed between sexes across 43 GTEx tissues by Oliva et al. (Oliva et al., 2020), there were 58 genes identified close to these 12 SNPs, among which 34 genes adjacent to eight SNPs displaying sex-biased differential expression at least in one of 43 GTEx tissues (Figure 2). Details of these eight SNPs mapped to 34 sex-biased genes are included in Table 1. They are rs1134004 (*S1PR3*, *GADD45G*, *SHC3*, *SECISBP2*, *SEMA4D*, and *C9orf47*), rs140657166 (*TRIM29*, *OAF*, *THY1*, *PVRL1*, and *AP000679.2*), rs148143613 (*C10orf91, PWWP2B*, *KNDC1*, *LRRC27*, *STK32C*, and *INPP5A*), rs1965385 (*BASP1*), rs2443615 (*RRM1*, *STIM1*, *PGAP2*, and *TRIM21*), rs2924725 (*CSMD1*), rs35239301 (*SCHIP1*), rs555336963 (*GRM7*), and rs8116534 (*RBBP9*, *DZANK1*, *ZNF133*, *CSRP2BP*, *RRBP1*, *MGME1*, *OVOL2*, *POLR3F*, and *SEC23B*). Among these tissues displayed significantly sex-biased DGE, there are nine genes (*SEMA4D*, *STK32C*, *INPP5A*, *BASP1*, *RRM1*, *PGAP2*, *CSMD1*, *SCHIP1*, and *CSRP2BP*) showing constantly, slightly higher expression [log2 (fold change) between 0 and 1] in females, while other six genes, including *S1PR3*, *OAF*, *TRIM29*, *PVRL1*, *KNDC1*, and *STIM1*, display opposite effect sizes across different tissues between sexes. For example, both the expression of *PVRL1* and *TRIM29* are higher in females than in males among artery-tibial, artery-coronary and artery-aorta, with the expression of *TRIM29* is higher in skin not sun exposed suprapubic of females and the expression of *PVRL1* is higher in pituitary of females; additionally, lower expression of *PVRL1* is observed in female tissues of whole blood, brain-caudate basal ganglia, brain-hippocampus, brain-putamen basal ganglia, brain-nucleus accumbens basal ganglia, and muscle skeletal; in contrary, higher expression of *TRIM29* is observed in male adipose subcutaneous and muscle skeletal compared to that of female. Other 15 sex-biased genes display slightly lower expression [log2 (fold change) between −1 and 0] in females compared to males (see effect sizes and fold changes of these genes in Figures 2A,B). These genes are *GADD45G*, *SHC3*, *SECISBP2*, *THY1*, *AP0000679*.2, *C10orf91*, *TRIM21*, *DZANK1*, *RBBP9*, *ZNF133*, *OVOL2*, *RRBP1*, *MGME1*, *POLR3F*, and *SEC23B*, among which the expression of *TRIM21* is only significantly lower in cell cultured fibroblasts of females. Surprisingly, when comparing the expression of these genes in blood samples from COVID-19 patients with healthy controls in the COVID-19 expression database COVID19 db (Thair et al., 2021; Zhang et al., 2022), we found eight genes adjacent to five SNPs, including rs1134004 (*GADD45G*), rs140657166 (*TRIM29* and *PVRL1*), rs148143613 (*KNDC1* and *STK32C*), rs2443615 (*PGAP2* and *TRIM21*), and rs2924725 (*CSMD1*), are DEGs between blood of COVID-19 patients and healthy controls (multiple adjusted significance *p* < 0.015; one-way ANOVA test; Figure 2C). Collectively, our integrative analyses strongly support the potential involvements of these eight genes adjacent to five sex-biased COVID-19 SNPs in COVID-19 hospitalization.

## Discussion

By calculating the ΔZ-score of each SNP in the summary statistics of the sex-stratified COVID-19 hospitalization GWASs, we identified 12 top SNPs showing suggestive, sex-biased differential associations with COVID-19 hospitalization between males and females with European ancestry. Among these 12 COVID-19 SNPs, we found five SNPs have eight adjacent genes that are DEGs between sexes and up- or down-regulated gene expression in blood samples from COVID-19 patients when compared with healthy controls. These eight genes include *PVRL1*, *TRIM29, TRIM21*, *GADD45G*, *CSMD1*, *KNDC1*, *PGAP2*, and *STK32C*.

Previous studies (Lopez et al., 1995; Takekawa and Saito, 1998; Warner et al., 1998; Kraus et al., 2006; Higgs et al., 2008; Hansen et al., 2013; Dempster et al., 2014; Zhang et al., 2014; Kimura et al., 2015; Hayashi et al., 2017; Starnawska et al., 2017; Dou et al., 2019; Jones et al., 2021; Thair et al., 2021; Zhang et al., 2022) related to these eight genes strongly suggest their potential involvements in COVID-19. Among them, the most promising genes are *PVRL1, TRIM29,* and *TRIM21,* which are all located on chromosome 11*.* Interestingly, *PVRL1* and *TRIM29* are both close to the sex-biased COVID-19 association SNP rs140657166, and *TRIM29* is adjacent to rs2443615, with the two SNPs 115.5-Mbp far away from each other. According to GeneCards (Safran et al., 2010), *PVRL1* encodes a protein involved in the organization of adherents junctions and tight junctions in epithelial and endothelial cells, and it is also reported as a receptor for herpes simplex virus 1, herpes simplex virus 2, and pseudorabies virus (Lopez et al., 1995; Warner et al., 1998). In our DGE analysis between sexes, *PVRL1* is highly expressed in multiple tissues of females, including artery-tibial, artery-coronary, and artery-aorta (see Figures 2A,B). Furthermore, *PVRL1* was down-regulated in the blood samples of COVID-19 patients according to the COVID-19 expression database COVID-19db (Figure 2C). Interestingly, *TRIM29,* which is close to *PVRL1*, shows higher expression in most female tissues, including artery-tibial, artery-coronary, artery-aorta, and skin not sun exposed suprapubic, except for adipose subcutaneous and muscle skeletal where the expression of *TRIM29* is higher in male and *TRIM29* is up-regulated in blood samples of COVID-19 patient compared to healthy controls. TRIM29 is involved in IFN-γ signaling and cytokine signaling by playing an essential role in activating macrophage upon viral or bacterial infections within the respiratory tract (Li et al., 2018; Dou et al., 2019). Additionally, we found *TRIM21* is another potential target, the expression of which is only higher in cell cultured fibroblasts of males than that of females. *TRIM21* encodes an E3 ubiquitin-protein ligase, which plays key roles in immune host defense, signal transduction, and possibly cell cycle regulation (Jones et al., 2021). Specifically, TRIM21 is involved in multiple immune responses, including the negative regulation of IFN-β production post-pathogen recognition (Higgs et al., 2008), the promotion of IRF8 ubiquitination that subsequently enhances the ability of IRF8 to stimulate cytokine gene transcription in macrophages (Jones et al., 2021), and the attenuation of type I IFN-dependent immune responses (Kimura et al., 2015). Taken together, all pieces of evidence strongly support the involvement of *PVRL1*, *TRIM29*, and *TRIM21* in COVID-19, but further investigation is warranted.

In our integrated analyses, we also revealed other five candidate genes potentially involved in COVID-19, including *GADD45G*, *PGAP2*, *CSMD1*, *KNDC1*, and *STK32C*. In the sex-biased DGE analysis, we revealed that the expression of *GADD45G* and *PGAP2* are specifically higher across multiple GTEx tissues of males and females. *CSMD1* only shows significantly higher expression in female muscle skeletal than that of male. The three genes are all down-regulated upon SARS-CoV-2 infection in blood samples of COVID-19 patients. Another two genes, *KNDC1* and *STK32C*, adjacent genes for the same SNP rs148148613, tend to be expressed higher in most of GTEx tissues of females than males, and they are stimulated in blood of COVID-19 patients. Further functional annotations for these genes also support their potential involvements in COVID-19. Among these genes, *GADD45G*, a stress and DNA-damaging responsive gene, encodes the protein GADD45G that is a mediator in activating the p38/JNK pathway *via* MTK1/MEKK4 kinase and consequently regulates cell growth and apoptosis (Takekawa and Saito, 1998). *CSMD1* encoded protein CSMD1 is suggested to affect learning or memory, mammary gland branching during pregnancy, and development of reproductive structure (Kraus et al., 2006). The protein PGAP2 encoded by the gene *PGAP2* influences the maturation of glycosylphosphatidylinositol (GPI) anchors on GPI-anchored proteins, and protein coding mutations of *PGAP2* were suggested to lead to an autosomal recessive syndrome characterized as hyperphosphatasia and intellectual disability (Hansen et al., 2013). KNDC1 is encoded by the gene *KNDC1*, and it is a Ras guanine nucleotide exchange factor that was suggested to be involved in the dendritic growth in the brain (Hayashi et al., 2017); it was also reported to be involved in aging *via* its roles in the senescence of umbilical vein endothelial cells (Zhang et al., 2014). *STK32C* encodes the protein STK32C that is a member of the serine/threonine protein kinase family. According to GeneCards (Safran et al., 2010), *STK32C* is known to be highly expressed in the human brain, with highest expression levels reported in the cerebellum and frontal cortex. Further studies displayed that the top differentially methylated probes were located within *STK32C* and hypomethylation of a CpG site in an intron of *STK32C* in individuals may contribute to depression disorder (Dempster et al., 2014; Starnawska et al., 2017). Furthermore, *STK32C* is involved in sweet taste signaling. Given their important functions in multiple tissues and sex-biased expression patterns in specific tissues, the interrupted expressions of these five genes upon SARS-CoV-2 infection may lead to different COVID-19 symptoms between sexes. This may be especially true for *KNDC1* and *STK32C*, both of which are important to brain functions and show higher expression in female brain tissues; the interrupted expressions of the two genes may contribute to long COVID in a sex-biased manner. Taken together, the involvement of these five genes in COVID-19 hospitalization are needed for further investigations.

Our study has potential limitations. One limitation is the potential influence of the interaction between age and sex on the sex-biased COVID-19 association signals in the current study. However, the summary statistics of sex-stratified COVID-19 GWASs were generated by including age and 10 principal components as covariates. Determining the interaction of SNPs with sex and age on COVID-19 hospitalization requests us to perform COVID-19 GWAS from scratch by having raw genotyping data and clinical information, i.e., sex and age. It is well known that even larger sample sizes for both female and male GWASs are needed to detect the potential interactions between SNP and age / sex or age + sex. The sample sizes used by the current two COVID-19 hospitalization GWASs are underpowered to detect the above interactions. Another limitation is that these eight candidate genes display rather small fold change [absolute log2 (fold change) < 1] between sexes, and they were selected because of their close distances to 5 SNPs showing sex-biased associations with COVID-19 hospitalization. Specifically, we found the expression of *TRIM21* is relatively low with median TPM < 1 in muscle skeletal, although in which *TRIM21* demonstrates significant, sex-biased differential expression. We thus conclude that sex-biased differentiation of a gene need to be interpreted with caution if the gene showing lower expression in a specific tissue, especially when there are outliers with extremely high expression. The final limitation is about the re-analyzing of RNAseq data of blood of COVID-19 patients and healthy controls, in which we performed DGE analysis for genes adjacent to the candidate COVID-19 SNPs. Due to the relatively small sample size of the RNAseq data set and the unavailability of sex, age, and other clinical information for these samples, we could not exclude the potential effects of these confounding factors on the DEGs between COVID-19 patients and healthy controls, particularly for these candidate genes. We realize that it is necessary to conduct DGE analysis by including the interaction between sex and COVID-19 status, as well as other clinical information, into a linear regression model. If the interaction of sex and COVID-19 status for a candidate gene is significant, it would further support the potential involvement of the candidate gene in COVID-19.

In summary, our integrative comparison of male- and female-separated COVID-19 hospitalization GWASs provides new insights into the potential sex differences in COVID-19 hospitalization. Although no genome-wide significant SNPs showing differential COVID-19 associations between males and females, by conducting eQTL and DGE analysis between sexes among multiple GTEx tissues, and DGE analysis of blood samples between COVID-19 patients and healthy controls, we prioritize five SNPs with suggestive sex-biased differential association with COVID-19. We highlight eight genes close to these five candidate SNPs representing differential COVID-19 hospitalization associations between males and females. These genes, especially for *PVLR1*, *TRIM21*, and *TRIM29*, are strongly related to immune responses to infection, which are warranted for further cellular and animal studies.

## Data Availability

The datasets presented in this study can be found in online repositories. The names of the repository/repositories and accession number(s) can be found in the article/Supplementary Material.
