# Supplementary figures and images for: Genome-wide screening of sex-biased genetic variants potentially associated with COVID-19 hospitalization
Source: Front Genet. 2022 Oct 24;13:1014191. doi: 10.3389/fgene.2022.1014191 (PMC9637711; doi:10.3389/fgene.2022.1014191)

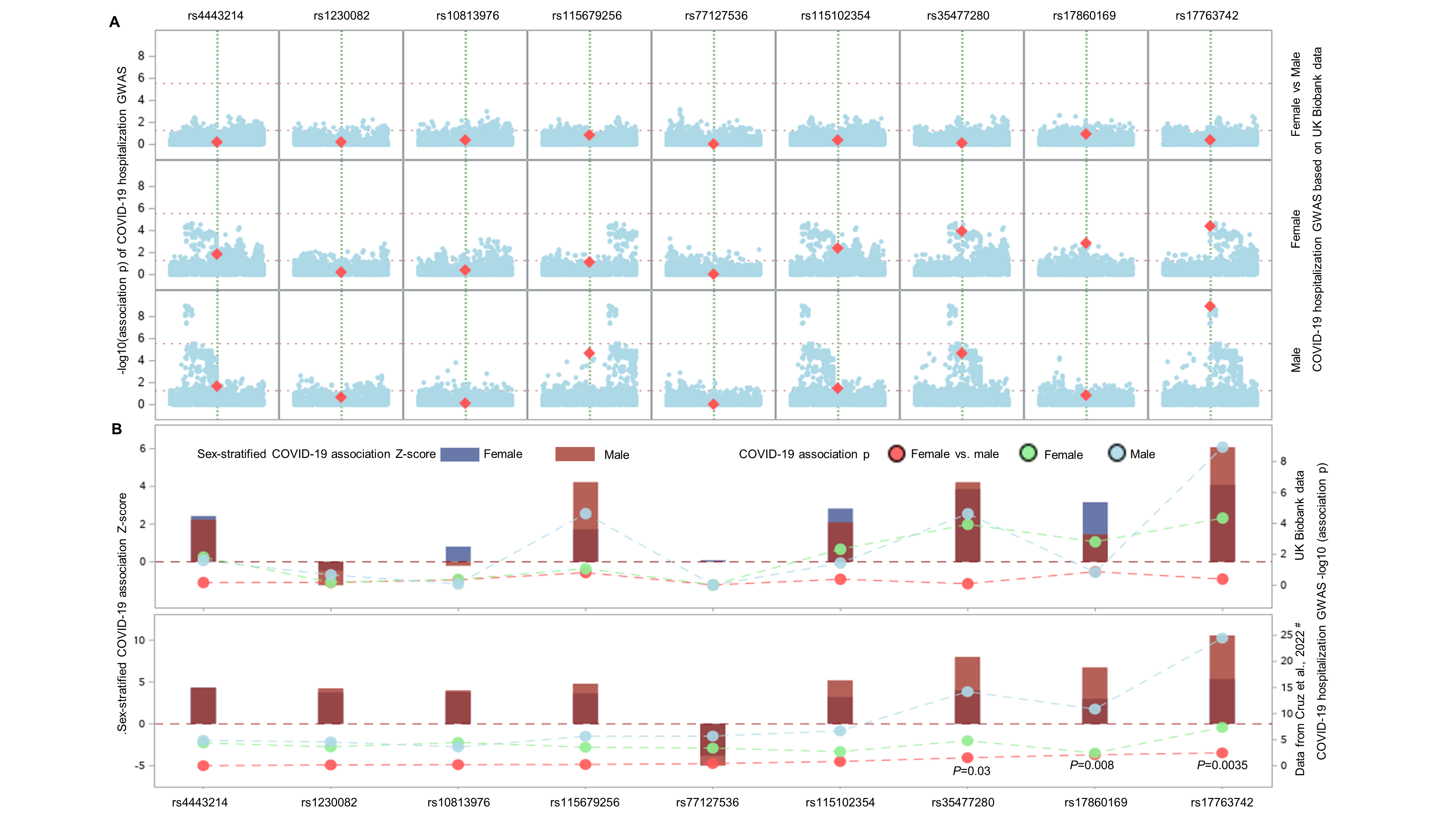

Supplement: Supplementary file 1 [file Image1.tif]
