# Supplementary material for: Genome-wide screening of sex-biased genetic variants potentially associated with COVID-19 hospitalization
Source: Front Genet. 2022 Oct 24;13:1014191. doi: 10.3389/fgene.2022.1014191 (PMC9637711; doi:10.3389/fgene.2022.1014191)
Supplement: Supplementary file 2 [file DataSheet1.pdf]

Figure S1. Evaluation of previously published COVID-19 risk SNPs in current sex-stratified and sex-biased COVID-19 hospitalization GWASs based on data from UK Biobank.

(A) Local Manhattan plot covering a 1-Mbp region harboring each of nine COVID-19 risk SNPs (highlighted in dark yellow color) that were published by (Cruz et al., 2022). Data used to generate the figure were from current sex-stratified and sex-biased COVID-19 hospitalization GWASs from UK Biobank. The first panel labeled as 'Female vs Male' demonstrates the ΔZ-score p values of SNPs located on the 1-Mbp region for each published COVID-19 risk SNP, and the lower two panels display the corresponding sex-stratified COVID-19 association signals in females and males separately. ΔZ-score was generated by comparing effect sizes of each SNP between females and males. A suggestive significance of ΔZ-score p-value threshold was set at $p < 5 \times 10^{-6}$, indicated by the upper red dashed line. There are 6 out of 9 published SNPs showing nominally significant associations with COVID-19 hospitalization in either females or males from UK Biobank, among which one SNP rs17763772, mapped to the gene cluster of *SLC6A20*, *LZTFL1*, *CCR9*, *FYCO1*, *CXCR6*, and *XCR1*, is genome-wide significant ($p < 5 \times 10^{-8}$) in the current UK Biobank COVID-19 GWAS of males. However, none of these SNPs displays significant, sex-biased associations with COVID-19 hospitalization in the UK Biobank GWASs. (B) Detailed comparison of Z-score, association p values, and ΔZ-score p values for these 9 published COVID-19 risk SNPs based on data from UK Biobank (upper panel) and published by (Cruz et al., 2022) (lower panel), are demonstrated with needle plots. The left y-axis illustrates the sex-stratified COVID-19 association z-scores, while the right y-axis displays the p values of sex-stratified associations and ΔZ-scores. The sex-stratified SNP Z-scores are illustrated with different color bars according to the left y-axis for both sexes (female: dark blue bar; male: dark red bar). In terms of association -log10(P) of these SNPs, dots with different colors are used to represent them, i.e., light green dots for female association signals, light blue dots for male association signals, and red dots representing p values of ΔZ-scores, are displayed in accordance to the right y-axis. To separate these p values and visualize the differences of Z-scores and association p values in both sexes, dashed lines are utilized to link and separate these dots representing association p values derived from sex-stratified analysis and ΔZ-score p values generated by differential Z-test between sexes. Three SNPs showing nominal significance of ΔZ-scores are provided with ΔZ-score p values in the figure, with other SNPs are all not significant in terms of ΔZ-scores.

Cruz, R., Almeida, S.D., Heredia, M.L., Quintela, I., Ceballos, F.C., Pita, G., Lorenzo-Salazar, J.M., González-Montelongo, R., Gago-Domínguez, M., Porras, M.S., *et al.* (2022). Novel genes and sex differences in COVID-19 severity. Human molecular genetics *ddac132*.
